# Discontinuity of Human Presence at Atapuerca during the Early Middle Pleistocene: A Matter of Ecological Competition?

**DOI:** 10.1371/journal.pone.0101938

**Published:** 2014-07-23

**Authors:** Guillermo Rodríguez-Gómez, Ana Mateos, Jesús Angel Martín-González, Ruth Blasco, Jordi Rosell, Jesús Rodríguez

**Affiliations:** 1 Paleofisiología y Ecología Social de homínidos, Centro Nacional de Investigación sobre la Evolución Humana (CENIEH), Burgos, Spain; 2 Departamento de Matemáticas y Computación, Universidad de Burgos, Burgos, Spain, and temporarily assigned to CENIEH, Burgos, Spain; 3 The Gibraltar Museum, Gibraltar, Gibraltar; 4 Àrea de Prehistòria, Universitat Rovira i Virgili (URV), Tarragona, Spain; 5 Institut Català de Paleoecologia Humana i Evolució Social (IPHES), Tarragona, Spain; University of Florence, Italy

## Abstract

Increasing evidence suggests that the European human settlement is older than 1.2 Ma. However, there is a fierce debate about the continuity or discontinuity of the early human settlement of Europe. In particular, evidence of human presence in the interval 0.7−0.5 Ma is scarce in comparison with evidence for the previous and later periods. Here, we present a case study in which the environmental conditions at Sierra de Atapuerca in the early Middle Pleistocene, a period without evidence of human presence, are compared with the conditions in the previous period, for which a relatively intense human occupation is documented. With this objective in mind, the available resources for a human population and the intensity of competition between secondary consumers during the two periods are compared using a mathematical model. The Gran Dolina site TD8 level, dated to 0.7−0.6 Ma, is taken as representative of the period during which Atapuerca was apparently not occupied by humans. Conditions at TD8 are compared with those of the previous period, represented by the TD6-2 level, which has yielded abundant evidence of intense human occupation. The results show that survival opportunities for a hypothetical human population were lower at TD8 than they were at TD6-2. Increased resource competition between secondary consumers arises as a possible explanation for the absence of human occupation at Atapuerca in the early Middle Pleistocene.

## Introduction

The current paleoanthropological scenario shows an increasingly complex human population dynamic for the Pleistocene period. Some phenomena, such as migrations, replacements of species and abandonment of territories with unfavourable conditions for human colonization, seem to have played significant role in the evolution of the genus *Homo*. From this perspective, one of the most interesting subjects is the continuity or discontinuity between the hominins of the Early and Middle Pleistocene in Western Europe and the origin of the Neandertal lineage [1]. This topic has been treated from different perspectives and by different disciplines. Several authors have proposed the existence of migration waves during the Early Pleistocene taking into account different evidence [2]–[13]. Bermúdez de Castro and Martinón-Torres [14] suggest a theoretical evolutionary scenario for the Early Pleistocene based on isolation processes and intermittent contacts between the Eurasian populations, as previously proposed by Dennell et al [15] for the Middle Pleistocene. According to their hypotheses, the hominins from Eurasia lost contact with Africa after the first *Out of Africa* because of the formation and evolution of significant biogeographical barriers between the two continents, such as the Negev desert [16], [17]. From this point forward, Eurasian populations followed an independent evolutionary process in which the same phenomena of climatic and geographical barriers caused periods of disconnection between some regions [13], [18]. Apparently, these frontiers mainly affected the European populations, producing alternating episodes of isolation with population fluxes and refluxes. This dynamic seems to have also occurred during the Middle Pleistocene and may help to explain some significant changes that occurred in Western Europe during this period. An alternative explanation relates human dispersal in Europe to climate change [12] and links the permanence of humans Oldowan technology in Europe to faunal continuity, and specifically to the presence of the sabertooth *Megantereon whitei*, a hypercarnivorous predator which, presumably, provided hominins with large amounts of scavengable carcasses [13]. Arribas and Palmqvist [13] suggest that there was isolation between Europe and Africa between 1.8 Ma until 0.6 Ma, when Achelean populations expanded from Africa into Western Europe.

Currently, human settlement during the Early Pleistocene is represented in Western Europe by a short list of sites, such as Fuente Nueva 3 [19], [20], Barranco León [21], Vallparadís [22], [23] but see also [24], Sima del Elefante and Gran Dolina in Spain [25], Pirro Nord [26] and Ca'Belvedere di Monte Poggiolo [27] in Italy, Le Vallonet [28], Saint-Hilaire-la Gravelle [29], [30] and Pont de Lavaud [31] in France, Happisburgh [32] in England and Untermassfeld [33] and Dorn-Dürkheim 3 [34] in Germany. The site of Pakefield (England), which dates to the beginning of the Middle Pleistocene (0.7 Ma) [35], can also be considered in this list. Lézignan le Cèbe, in southern France, has been proposed as one of the oldest sites documenting human occupation in Europe [36], but this interpretation is controversial. Technologically, all of these archaeological locations can be classified as Oldowan Technological Complexes or Mode 1 [11], [37]. *Homo antecessor*, described from level TD6 of Gran Dolina, seems to have inhabited this area at least between the Jaramillo Subchron and the Matuyama-Brunhes Boundary [38]. Human remains older than the Jaramillo Subchron have been found at Sima del Elefante (1.2 Ma) [39] and Barranco León (1.4 Ma) [21], but see also Muttoni et al. [40]. Unfortunately, these fossil remains do not meet enough taxonomic criteria to establish possible relationships with other species such as *H. antecessor* and to describe the human scenario at this early time. In the absence of other data, these fossils have been classified as *Homo* sp. [21], [38].

Mosquera et al. [11] postulated a general continuity in the hominin occupation of Sierra de Atapuerca with the exception of a gap between 0.9 and 0.5 Ma at the Gran Dolina site, based on the archaeological evidence. These authors suggest that this depopulation was not a local phenomenon but a European event. During this period, evidence of human presence is scarce in Western Europe. Although no human remains have been recovered between OIS18 and OIS16, some isolated lithic assemblages have been dated to the beginning of this period, such as those found at the Middle Loire River Basin [41] and at Caune de l'Arago [42] in France. In contrast, the number of archaeological sites increases significantly in approximately 0.6−0.5 Ma, revealing new palaeoanthropological and cultural features that mark the European Middle Pleistocene [12], [43], [44]. New lithic assemblages with early Acheulean industries seem to appear in Western Europe at the end of the Early Pleistocene ([11] and reference therein) although the dates of some sites are debatable [12]. In any case, Acheulean technology arrived in Europe around or before 0.6−0.5. Ma. The new settlers, with full Acheulean Mode 2 technology, occupied the European landscapes intensely at this time, as shown by the increasing number of sites. The sparsely populated northern latitudes began to be systematically occupied. Examples of this phenomenon can be found at Boxgrove in England [45], and at Bilzingsleben [46] and Schöningen [47] in Germany. Interestingly, the oldest European hearths have been dated to approximately 400 ka (Terra Amata, Beeches Pit, Schöningen or Bilzingsleben) [11], [48]. The hominin fossil evidence is currently the focus of an intense debate concerning the evolutionary relationships of the Early and Middle Pleistocene European hominins [1], [15], [49]–[53] and references therein.

Given the shortage of sites and the sparse anthropic evidence from the beginning of the European Middle Pleistocene, three possible scenarios may be considered. In the first scenario, a discontinuity occurred between the Early and Middle Pleistocene human populations, and thus, Europe was almost depopulated at this time. Alternatively, if continuity between the Early and Middle Pleistocene human populations occurred, the scarcity of evidence might be explained by a contraction of the human populations as a response to adverse environmental conditions. In this latter case, the Early Pleistocene hominins would have survived in some refuges where the environment remained more consistent and eventually evolved into the new Middle Pleistocene forms, both anatomical and culturally. A third possible scenario is that the Early Pleistocene human populations survived in some refuges from OIS18 to OIS14 but were eventually substituted by the Acheulean immigrants approximately 0.5 Ma. Alternatively, it could be argued that the scarcity of evidence of human presence is just a consequence of the low completeness of the fossil record. Although this possibility may not be definitively ruled out, the scarcity of lithic record in this period, in comparison to the previous one, is striking, since stone tools are abundantly produced by humans and they have a high probability of being preserved in the archaeological record.

Here, we focus on the critical period 0.7−0.5 Ma to test whether the lack of evidence of human presence is related to adverse ecological conditions during this period. Resource availability and competition with carnivores have been repeatedly proposed as key limiting factors for Early Pleistocene European hominin populations [4], [13], [54]–[59]. Thus, a key question is whether resource availability was lower in the period between OIS18 and OIS12 than at the end of the Early Pleistocene. Rodríguez-Gómez et al. [60] presented a model to study predator-prey relationships and estimate resource availability on a local scale. This model is used here to test the hypothesis that the absence of human settlement was coincident with a period of low resource availability.

The Gran Dolina site in the Sierra de Atapuerca (in northern Spain) provides a unique opportunity to test this hypothesis on a local scale. This site has a long stratigraphic sequence that dates from the Jaramillo Subchron to the end of the Middle Pleistocene. Evidence of human presence at Sierra de Atapuerca before and after the critical period 0.7−0.5 Ma has been registered at different sites and stratigraphic levels. Human settlement in the 1.2−0.7 Ma period is documented at the TE9 level at Sima del Elefante, and the TD3-TD4 and TD6 levels at Gran Dolina, while the GII, GIIIa, GIIIb units of Galería, the TD10-2 and TD10-1 levels of Gran Dolina and the Sima de los Huesos site document human settlement during the period 0.5−0.25 Ma [61]–[64]. The interval between OIS18 and OIS16 (roughly from 0.7 to 0.5 Ma) is represented at Atapuerca by the TD7, TD8 and TD9 levels of Gran Dolina, all of which lack evidence of human presence in the Atapuerca area [25]. This archaeological gap in Atapuerca has been discussed taking as a perspective the technological evidence [11], [43]. The TD8 level provides the richer faunal assemblage of this period at Atapuerca [62], [63]; thus, it has been selected for the study of this critical period.

The aim of this study is to compare competition intensity and resource availability at Atapuerca during the period for which evidence of human presence is lacking, represented by the TD8 assemblage, with the conditions at the end of the Early Pleistocene, represented by the TD6-2 assemblage, a period of intense human occupation at Atapuerca. We introduce in this analysis an index of intraguild carnivore competition to compare the changes in the local conditions between these two periods. These data will provide us which information about the main features of the palaeoecosystem and the survival opportunities for a hypothetical human population at the Sierra de Atapuerca during this period.

## Materials and Methods

### The Atapuerca Gran Dolina TD8 Level

The Sierra de Atapuerca is located 15 km east of Burgos, in the north of the Iberian Peninsula. This Cretaceous limestone massif has various cavities filled with well-stratified Pleistocene sediments, known from south to north as Sima del Elefante (TE), Galería (G) and Gran Dolina (TD). These sites have been excavated since 1981 by Atapuerca Team with permission of the Consejería de Cultura y Turismo de la Junta de Castilla y León. Gran Dolina (TD) (3°31′08 W, 42°21′09 N; UTM coordinates: X = 457279, Y = 4689172) is a cavity approximately 18 m in height that was filled with Lower and Middle Pleistocene sediments. Its stratigraphic sequence was initially divided into 11 stratigraphic levels (TD1-TD11 from bottom to top) and revised in subsequent studies; e.g., [25].

The TD8 level was formed by a succession of brecciated flows of red lutites with boulders and gravels [65]. Its sedimentation is composed of overlapping cones with main vertexes at the north of the site. The vertical positions of these vertexes and their proximity to the roof suggest a small entrance into the cave during the TD8 formation. The ESR and U-series dates taken from the middle part of the sedimentary deposit suggest an average age of 600 kyr (602±52 kyr) [63]. The result obtained by TL from one sample collected at the base of TD8 correlates with the Matuyama-Brunhes boundary when the range of error is taken into account (820±140 kyr) [62]. From an archaeological perspective, evidence of human presence was not documented at TD8, as noted above. It might be argued that the small entrance and the general conditions (humidity and darkness) of the cave prevented a continuous access by a hypothetical human population, although the evidence available is not conclusive on this respect.

Taking into account the species of carnivores recovered at the TD8 level (see below) and the taphonomic characteristics identified in the faunal assemblage (e.g., tooth-mark dimensions), hyena is proposed as the main agent responsible for the ungulate accumulations [66]. However, a certain degree of variability with respect to those features traditionally used to define carnivore dens can be observed at TD8 (e.g., an absence of immature carnivore remains, a low proportion of coprolites, few marks related to the end stages of carnivore consumption, the absence of an attritional age profile and large quantities of whole bones and epiphyses) (see [67]). According to Blasco et al. [66], this variability seems to be the result of a combination of several types of dwellings (dens and refuges) and the occasional access of other carnivores during the formation of the TD8 deposit.

### The TD8 Faunal assemblage

The faunal list for TD8 was obtained from Rodríguez et al. [25], Blasco et al. [66], and van der Made [68]. We restricted our analysis to mammal species of weighing more than 10 kg because these include the main sources of meat and fat, as well as the main potencial competitors and predators of a hunter-gatherer population [69], [70]. This size interval includes small to medium-sized predators such as the lynx that may prey on small ungulates that are potentially important in a human diet. Rodríguez [71] provides estimations for the body mass of the mammal species identified at Gran Dolina based on the few fossils available at that time and using allometric equations. However, excavation of Gran Dolina in subsequent years increased the fossil sample from TD8. Gran Dolina TD8 site contains 899 remains that belong to mammal species of more than 10 kg. No specific permits are required for this study, which complied with all relevant regulations. The current regulation is Decreto 37/2007, 25th of April 2007, Junta de Castilla y León Cultural Heritage (BOCyL 79, 25/05/2007). The material revised is temporary housed at Institut Català de Paleoecologia Humana i Evolució Social (IPHES) at Tarragona (Spain). This research is under the frame of the Research Project CGL2012-38434-C03-02 from Spanish MINECO. The specimen numbers are provided in (Table S1).

Thus, we analysed the entire TD8 fossil assemblage and reviewed the body size estimations provided by Rodriguez [71], recalculating the body masses with allometric equations [72] when new data were available. There are eleven primary consumer species in TD8 weighing more than 10 kg: *Bison voigtstedtensis, Cervus elaphus* (red deer), *Dama vallonetensis, Equus altidens, Eucladoceros giulii, Hippopotamus* sp., *Macaca* sp., *Megaloceros solilhacus, Stephanorhinus etruscus, Sus scrofa* (wild boar) and *Ursus* sp. The bear, *Ursus* sp., is considered to be an omnivore, and in this study, it was included as both a potential prey and a potential predator. Five large predators were identified in the TD8 assemblage: *Canis mosbachensis, Crocuta crocuta* (spotted hyaena), *Hyaena* sp. (considered analogous to striped hyaena), *Lynx* sp., *Panthera gombaszoegensis and Ursus* sp. The small canid *Vulpes* sp. was not included in the analyses because its diet was presumed to be composed of small mammals, as discussed by Rodríguez-Gómez et al. [60]. The families Viverridae and Mustelidae were also excluded because their diets are also based mainly on small mammals. The TD8 fossil assemblage lacks adequate elements to estimate the body masses of *Hippopotamus* sp., *Hyaena* sp., *Macaca* sp., *Megaloceros solilhacus*, *Panthera gombaszoegensis* and *Sus scrofa* using allometric equations. Thus, for these species, we used the body masses provided by Rodríguez [71] and Blasco et al [66], except for *Hippopotamus* sp., for which the body mass for *Hippopotamus* gr. *H. antiquus* provided by Mazza and Bertini [73] was used. The body masses used are shown in Table 1.

**Table 1:** Estimated body masses of the species in the TD8 assemblage.

Species	Estimated Body Mass (kg)
*Bison voigtstedtensis*	397
*Cervus elaphus*	163
*Dama vallonetensis*	84
*Equus altidens*	324
*Eucladoceros giulii*	360
*Hippopotamus* sp.	2,225
*Megaloceros solilhacus*	383
*Macaca sylvanus*	18
*Stephanorhinus struscus*	1,400
*Sus scrofa*	85
*Ursus* sp.	282
*Canis mosbachensis*	12
*Crocuta crocuta*	75
*Hyaena* sp.	50
*Lynx* sp.	10
*Panthera gombaszoegensis*	90

Weights of *Hyaena* sp., *Macaca* sp., *Megaloceros solilhacus*, *Panthera gombaszoegensis* and *Sus scrofa* were taken from Rodríguez [71] and Blasco et al [66]. To *Hippopotamus* sp. weight was taken from [73].

Although we lack evidence of human presence at TD8, *Homo* sp. has been included in the analyses as a hunter-gatherer to estimate competition and resource availability for a hypothetical human population living in Atapuerca at that time. We assume for *Homo* sp. the same trophic behaviour inferred for *Homo antecessor* in the TD6-2 assemblage. Hunting has been interpreted as the main food procurement strategy at TD6-2 [74], [75], although other Early Pleistocene sites like Fuente Nueva-3 evidence a predominantly scavenging behaviour of early *Homo* [55].

Thus, we analysed the distribution of resources between secondary consumers for two different scenarios and examined the effects of these different configurations of the TD8 paleoecommunity: 1) a TD8 assemblage and 2) a TD8 assemblage + *Homo* sp.

Rodríguez Gómez et al. [60] used a mathematical model to quantify the resources available for a human population in the ecosystem represented at TD6-2. TD6-2 is another level at the Gran Dolina site, lately dating to approximately 0.9 Ma [61]. This level has yielded abundant faunal remains [25], [76], [77] and Mode 1 stone tools, together with a large assemblage of human fossils attributed to *Homo antecessor* [8]. The study by Rodríguez-Gómez et al. [60] on the TD6-2 assemblage, suggests the existence of a rich environment, abundant in trophic resources for a hominin population, at Atapuerca at the end of the Early Pleistocene.

To make the results for TD6-2 and TD8 directly comparable we also ran the model for TD6-2, including *Homo antecessor* as a secondary consumer, because it was not included in [60]. Two different levels of animal food in the diet of *Homo* were tested to represent either a diet with a low (30%, H_min_) or high (60%, H_max_) meat component. In addition, we included two scenarios for TD6-2, adding a large felid to its carnivore guild, although none was recorded in the fossil assemblage. Thus, we repeated our analyses, including *Homotherium latidens* as a member of the TD6-2 predatory guild, to evaluate the effect of its possible presence on our results, as explained in Rodríguez-Gómez [60].

### The model

**Figure 1:**
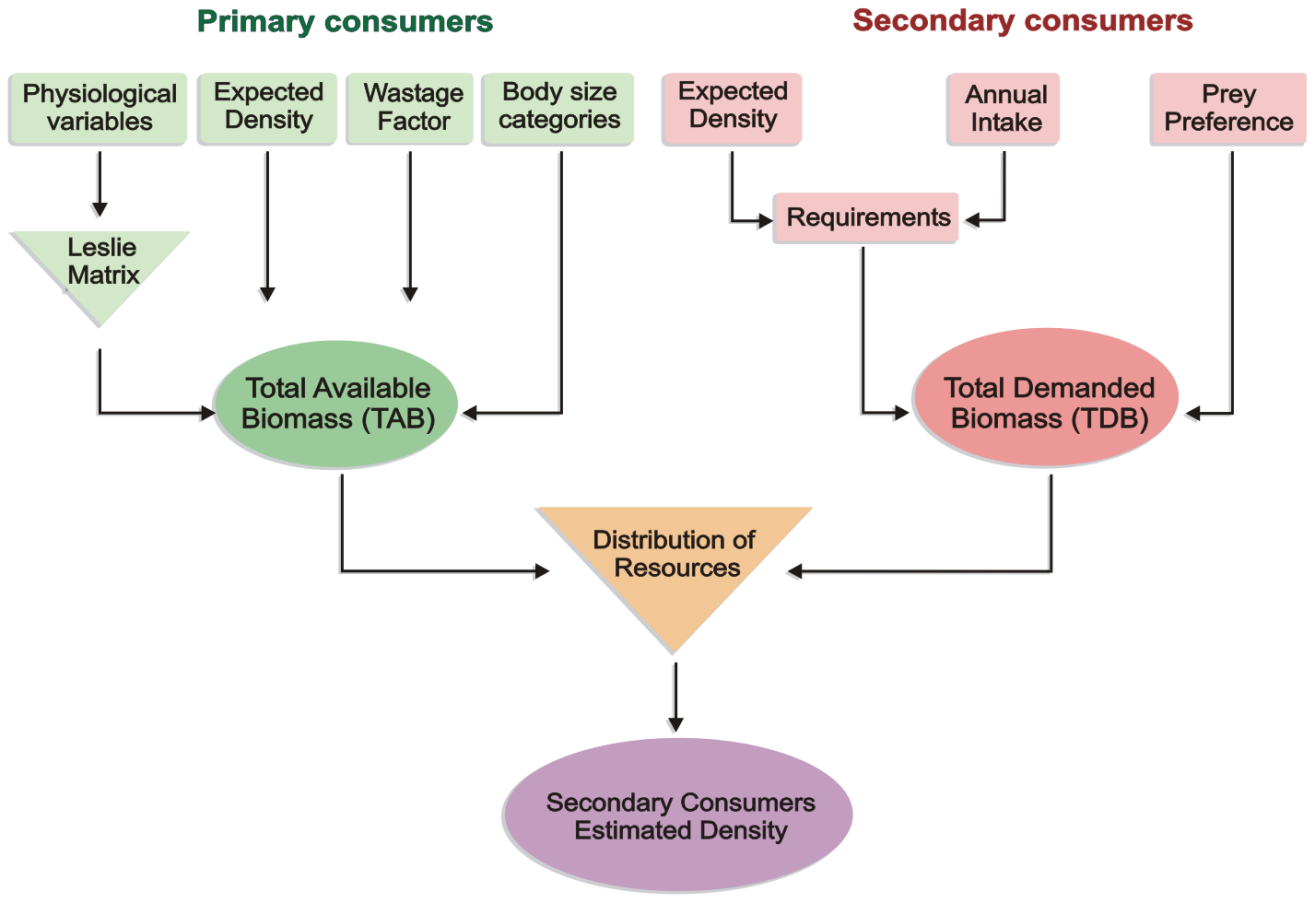
Flow diagram showing the components of the model used to evaluate trophic resource availability and intraguild competition (modified from [78]).

We investigated the distribution of resources, primary consumer biomass, among secondary consumers in different scenarios. On the one hand, it is necessary to estimate resource availability, i.e., the biomass of primary consumers available to secondary consumers or total available biomass (TAB). On the other hand, the requirements of secondary consumers (TBD) should also be estimated (Figure 1) [78]. A summary description of the model components is provided below; for a detailed formal description of this model see Rodríguez-Gómez et al. [60]. The model was written and executed in Matlab R2009b.

### Total Available Biomass

**Figure 2:**
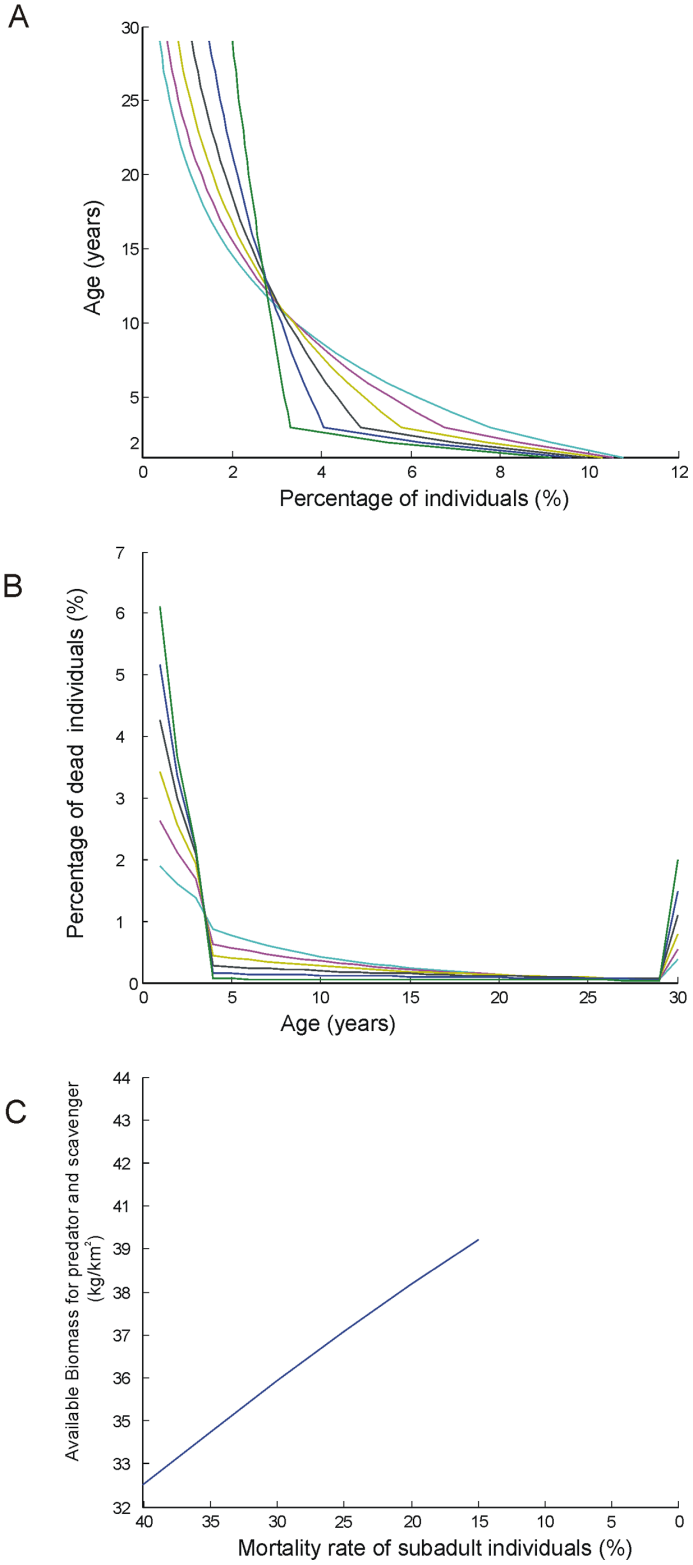
Graphical representation of population profiles (A), mortality profiles (B) and biomass output available for secondary consumers (C) at different sub-adult mortality rates for *Equus altidens*. Each line represents the solution of the model for a different level of sub-adult mortality. The percentage of dead individuals in the mortality profiles represents the percentage of individuals in the population that died at age *i*.

Our model was developed on the basis of the assumption that all of the variations in population size and composition may be taken as oscillations around a mean value that is constant through time-an assumption that is widely accepted in population dynamics studies [79]. We represented the average long-term condition of every population using a Leslie Matrix [80], [81]. Leslie Matrices are used in population dynamics to represent a population structure at different times and to describe its oscillations (Figures 1 and 2A). We sought the Leslie Matrix that represented the average structure over time by introducing two additional conditions:

- The population should be stable (i.e., population size should be constant from year to year).
- The population should be stationary (i.e., the age structure should be constant from year to year).

Input data are physical and physiological variables (adult body mass, body mass at birth, litter size, breeding interval, age at reproductive maturity, growth rate and lifespan) that are species specific. For species with living representatives, such as *Sus scrofa* and *Cervus elaphus*, the values of these physiological variables were taken directly from the literature. For species without living representatives, the values reported in the literature for closely related living species of similar body mass were used (e.g., *Equus zebra* was used as an analogue for *Equus altidens*). For species defined to genus level such as *Macaca* sp., we used values for species of similar size, as *Macaca sylvanus*. In the cases of *Bison voigtstedtensis*, *Dama vallonetensis*, *Eucladoceros giulii*, *Hippopotamus* sp., *Stephanorhinus etruscus* and *Ursus* sp., which lack closely related living species of similar size, we computed a least square regression equation for each physiological variable with respect to mean body weight using data for species in the same family; see further details in Supplementary Material, Table S3 in [60].

The population profiles obtained from this model for every primary consumer population provide estimates of the average sustainable biomass output by age classes, which were eventually translated into body size intervals. Biomass output by age interval was obtained from the annual mortality rates obtained from the Leslie Matrix (Figure 2B). Each dead individual of a primary consumer species was assigned to one of six size categories according to its average body mass at the age of death: 10–45 kg, 45–90 kg, 90–180 kg, 180–360 kg, 360–1,000 kg or >1,000 kg (see [57]). The biomass made available for secondary consumers by each single primary consumer population was obtained as the sum of the biomass of all dead individuals (Figure 2C).Total biomass output (TBO) was obtained as the sum of the biomass outputs in each size category from each primary consumer population (Figure 1). The distribution of sustainable TBO by size category is an important feature of the model, because prey body size is a main selection factor for predators [82]–[84]. With this treatment of the data it is then possible to represent features such as the hunting of sub-adults in species with very low adult mortality rates and very low predation at the adult stage, such as rhinos and hippos. This model may overestimate TBO because it considers that all the available biomass was consumed exclusively by mammals. The role of other secondary consumers, like vultures is not been considered in the model, although we acknowledge its relevance in actual ecosystems. There is not way to estimating the amount of TBO consumed by non-mammalian secondary consumers in the past, but it is reasonable to assume that it was similar in both levels (TD6-2 and TD8), an assumption that strengthens the null hypothesis of the absence of ecological differences between both assemblages.

Only female individuals are represented in a Leslie Matrix. Thus, we assumed the sex ratio was equal to 1∶1, that the population profile was the same for males and females and that the survival rate was equal for both sexes. A further assumption is that the sub-adult survival rate should be lower than the adult survival rate [60].

The model solutions are not dependent on population size: thus, an estimate of population density is needed to estimate the sustainable biomass output. Mediterranean taxa dominate the TD8 pollen spectrum [25]; thus, we used the equation provided by Damuth [85] to estimate the density of primary consumers in a European mixed temperate forest:
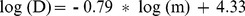
where D is the population density in number of individuals per square kilometre and *m* is the body mass in grams.

Combining the mortality profiles obtained from the Leslie Matrix with the mean body size per age class and the estimated population density of the species, the sustainable biomass output can be computed. Because a carcass includes a variable amount of non-edible tissues (horns, bones, hide, etc.), this sustainable biomass output cannot be fully used by secondary consumers. The percentage of non-edible TBO is represented in the model by a size-specific “wastage factor” [86]. Once this percentage has been subtracted, the final amount of biomass available to secondary consumers or total available biomass (TAB) is obtained. TAB is also distributed by body mass classes (Figure 1).

Our model yields several population profiles for each species, corresponding to different mortality rates. We selected extreme values with maximum and minimum pressure on sub-adults (or maximum and minimum mortality rates) that produce minimum and maximum TAB levels, respectively (TAB-m and TAB-M, respectively). We limited the results to those solutions for which sub-adult mortality rates were higher than adult mortality rates because this is the pattern usually observed in natural populations [87].

### Total Demanded Biomass (TDB)

Carnivore-demanded resources should be estimated as a first step in evaluating resource distribution among secondary consumers. The carnivore intake rate was estimated using the equation reported by Farlow [88]:
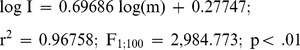
where *I* is the intake rate in kcal per day and *m* is the body mass in grams. Some adjustments were made for each secondary consumer according to its inferred dietary preferences. Taking the coyote as an analogue, we estimated that large mammal flesh represented only 20% of the energetic requirements of *Canis mosbachensis* and that it was primarily consumed as carrion [57]. The spotted hyena, *Crocuta crocuta*, is able to exploit bone marrow thanks to its bone-crashing abilities. We assumed that approximately 2% of the total energetic requirements of the spotted hyaena were satisfied by bone marrow, and consequently, its energetic requirements were reduced by a factor of 0.02 [89], [90]. In the case of *Hyena* sp., by analogy with the striped hyaena, carrion of large mammals would represent approximately 75% of its diet; the remainder could be invertebrates, vegetable food and small vertebrates [91]. The diet of the TD8 lynx (*Lynx* sp.), was presumed to be similar to that of the recent Iberian Lynx (*Lynx pardina*) [57]. Thus, it was assumed that species between 10 and 90 kg represented only 5% of the lynx diet [92]–[94], lagomomorphs and other small mammals making up the bulk of its diet. Because *Ursus* sp. was likely to have been highly omnivorous, it was assumed that meat represented only 10% of its energetic requirements as in recent European brown bears (*Ursus arctos*) [95], [96]. Likely, fish was also an important component of its diet as suggested by the nitrogen-isotope values obtained for the Venta Micena bears [97].

The annual energetic requirements of a carnivore population by km^2^ are obtained multiplying the individual annual intake by the population density (Figure 1). The equation provided by Damuth [98] for African flesh-eaters was used to estimate the typical carnivore density:
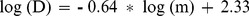
where *D* is the population density in number of individuals per square kilometre and *m* is the body mass in grams. In the case of *Ursus* sp., the equation for carnivores was used instead of the equation for primary consumers because it predicts the population densities of recent bear species more accurately [99]–[101].

The requirements of *Homo* sp. were estimated to be similar to those of recent hunter-gatherer populations, with a mean daily requirement of 3,000 kcal per individual [102]. The average body mass was considered to be 76 kg, based on pelvis and femur estimates for *Homo heidelbergensis* from the Sima de los Huesos site [103], [104]. Studies of recent hunter-gatherer populations have shown that animal resource consumption represents between 30% and 60% of their nutritional intake [105], [106]. Thus, we modelled *Homo* sp. as a species that meets 30% (H_min_) or 60% (H_max_) of its energetic requirements (3,000 kcal/day) by consuming the meat of large mammals. Population densities and diet compositions observed for recent hunter-gatherers populations are shown for comparison in Table 2 (extracted from [107]).

**Table 2:** Population densities and food resource use observed in recent hunter-gatherer populations [107].

Group	Area	Density ind./km^2^	Gathering%	Hunting%	Fishing%
Punan	29.6	0.118	65.00	30	5
Siberian Eskimo	274.9	0.047	1.00	30	60
Guayaki-Ache	28.7	0.0348	30.00	62	10
Efe	47.0	0.1596	88.20	11	0,8
Hadza	25.0	0.240	60.00	40	0
!Kung	110.0	0.066	67.00	33	0
G/Wi	180.0	0.0293	55.00	45	0
Mardudjara	226.0	0.0075	70.00	30	0
Kiowa	280.0	0.014	20.00	80	0
Caribou Inuit	2365.0	0.003	0.10	55	44.9
Nunamiut Inuit	249.0	0.0096	0.10	89	10.9
Polar Inuit	731.0	0.0041	0.01	30	69.99

As in the case of TAB, TDB was distributed over the same six body size categories based on the inferred prey size preferences of each predator, on the basis of the behaviour of their living relatives (Table 3 and Figure 1) [57]. The preference of a predator for a body size category is represented by the percentage of predation (PD) that this size category was presumed to represent in its diet. If a predator was presumed to be unable to kill prey in a given size category and to not consume carrion, a PD of 0 was assigned to the predator in that size category.

**Table 3:** Percentage of predation (PD) of the TD8 carnivores plus *Homotherium latidens* and *Homo* sp. by body mass category.

Species	Body size range (kg)					
	10–45	45–90	90–180	180–360	360–1,000	>1,000
*Canis mosbachensis*	17	17	17	17	17	17
*Crocuta crocuta*	21	32	26	11	5	5
*Homo* sp.	24	29	19	14	10	5
*Homotherium latidens*	6	6	28	33	22	6
*Hyaena* sp.	17	17	17	17	17	17
*Lynx* sp.	75	25	0	0	0	0
*Panthera gombaszoegensis*	6	6	31	38	19	0
*Ursus* sp.	17	17	17	17	17	17

The scores represent the relative importance that animals in each body size category had in the carnivore's diet. The sum of the scores in a row is 1. A score of 0 indicates no consumption of mammals in that body size category.

### Distribution of TAB between secondary consumers

The distribution of TAB between secondary consumers is based on the proportional predation pressure (PPP_ij_) of each species in each body size category. PPP_ij_ represents the relative amount of biomass demanded by the *jth* carnivore species from the *ith* prey body size category and is calculated as the proportion of the total amount of biomass demanded from a prey body size category by all carnivores that corresponds to the requirements of a single carnivore species. PPP_ij_ incorporates intraguild competition in the model because the resources obtained by a carnivore species depends on the number and characteristics of its competitors. See a detailed formal description of resource distribution computation in Rodríguez-Gómez et al. [60].

To compare the different scenarios considered in our analysis, we generated two indices (SCI and GCI) that relate the estimated and expected (estimated from allometric equations [98]) secondary consumer densities.

Species competition index (SCI):
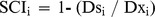
where *Dx_i_* is the expected density for species *i* obtained from the allometric equation in Damuth [98]. *Ds_i_* is the estimated density for species *i* and is obtained from our model.

Global competition index (GCI):
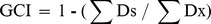
where *∑Ds* is the summation of estimated densities for all species and *∑Dx* is the summation of expected densities for all species.

These indices provide information about competition intensity in the ecosystem with regard to an ideal condition in which all species reach optimal densities. We assume that if the densities estimated from our model approach these thresholds densities, all species would fulfil needs with the have plenty of resources available to them. The SCI index shows to what degree a species fulfils its requirements in a given environment. GCI performs similarly but at the scale of the whole guild of secondary consumers guild. In both cases, however, if the estimated densities were closer to the expected densities or optimal ones, we considered that resources were abundant when compared with requirements (i.e. that competition is low). Conversely, if our model results indicate that the secondary consumers were able to coexist only at low densities, this would be so because there was a scarcity of resources in relation to requirements, which implies a high competition.

## Results

### Total Available Biomass

**Figure 3:**
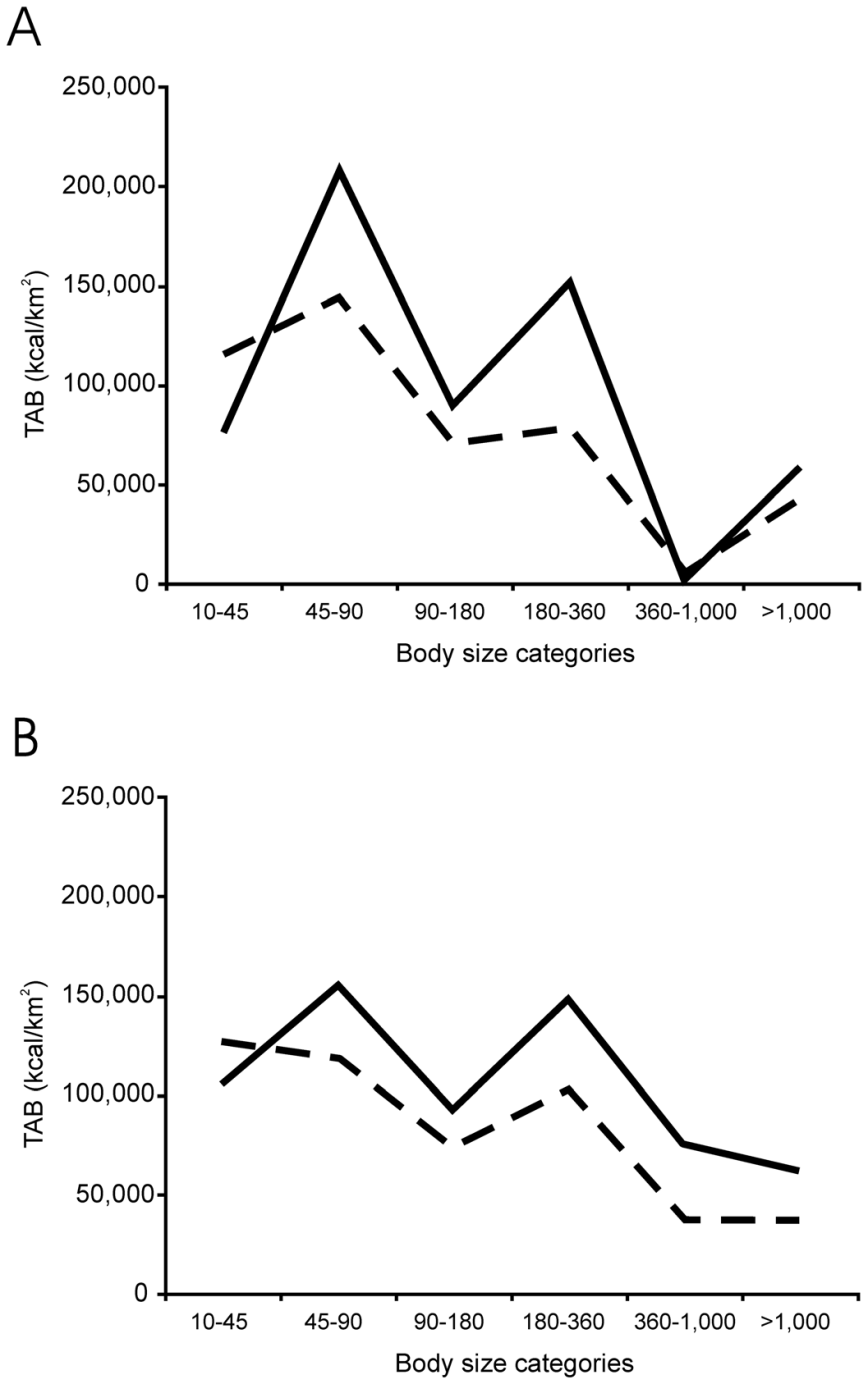
Biomass output distributed among six body size categories (see text). Dashed line: maximum sub-adult mortality rate for all primary consumer species or TAB-m; solid line: minimum sub-adult mortality rate (greater than or equal to the adult mortality rate) or TAB-M. A: TD6-2 available biomass; B: TD8 available biomass.

The minimum TAB, corresponding to the maximum sub-adult mortality rate, was 496,814 kcal/km^2^*year (TAB-m), and the maximum TAB, corresponding to the minimum sub-adult mortality rate, was 643,968 kcal/km^2^*year (TAB-M). TAB-m is 23% lower than TAB-M. In addition, the distributions of TAB-M and TAB-m by size category are different (Table 4 and Figure 3), mainly because TAB-m has more biomass in the first category (10–45 kg) than TAB-M. The reason for the different distribution is that both adults and sub-adults of *Macaca* sp. are present in this category and with a low sub-adult mortality rate, the biomass output of macaques is higher, increasing TAB in this size category. For the rest of species considered, adult and sub-adult individuals belong to different body mass categories, and biomass increases in larger body size categories as sub-adult mortality rate decreases. These results demonstrate the importance of including the age structure of primary consumer populations in the model, because the distribution of TAB in size categories is not homogeneous, and prey body size is a key factor in prey selection by most predators. Thus, the TAB of a prey size category is not equally available for all secondary consumers.

**Table 4:** Maximum and minimum total available biomass (TAB) in kcal/km^2^*yr, distributed over the six body size categories for the TD8 assemblage.

Body size range (kg)							
	10–45	45–90	90–180	180–360	360–1,000	>1,000	Total
TAB-M	106,512	155,722	92,136	149,351	76,849	62,630	643,201
TAB-m	126,928	118,768	72,589	102,942	38,128	37,304	496,659

The maximum TAB (TAB-M) is obtained when the sub-adult mortality rate is 0, while the minimum TAB (TAB-m) is obtained when all populations sustain the maximum possible sub-adult mortality rate while simultaneously satisfying the premises of stability and stationarity.

### Requirements of the secondary consumers

The energy necessary to maintain the species of the TD8 carnivore guild (first scenario) at their ecological densities is 693,725 kcal/km^2^*year (Table 5). This energy requirement exists if all species fulfil all of their expected requirements. The species with higher energetic requirements are *Crocuta crocuta* and *Panthera gombaszoegensis*, approximately 222,000 kcal/km^2^*year each, or 32% of total requirements. Next is *Hyaena* sp., which accounts for approximately 160,000 kcal/km^2^*year or 23% of the total. *Canis mosbachensis* accounts for approximately 40,000 kcal/km^2^*year or 6% of the total. *Lynx* sp. and *Ursus* sp. account for approximately 20,000 kcal/km^2^*year. However, these are estimates, and to establish whether all of the requirements of the secondary consumers are fulfilled, the distribution of TAB by size category and the prey preferences of the carnivores should be taken into consideration.

**Table 5:** Requirements of the secondary consumers in the TD8 assemblage plus *Homo* sp.

Species	W (kg)	D (ind/km^2^)	Requirements (kcal/km^2^/year)	Percentage requirement (%)	Percentage requirement with a less *Homo* sp. hunter (%)	Percentage requirement with a more *Homo* sp. hunter (%)
*Canis mosbachensis*	12	0.42	40,062	0.06	0.05	0.05
*Crocuta crocuta*	75	0.13	217,864	0.32	0.28	0.26
*Lynx* sp,	10	0.47	19,825	0.03	0.03	0.02
*Hyaena* sp,	50	0.17	162,932	0.24	0.21	0.19
*Panthera gombaszoegensis*	90	0.11	224,627	0.33	0.29	0.27
*Ursus dolinensis*	282	0.06	23,970	0.03	0.03	0.03
*Homo* sp. (30%)	76	0.24	78,840	**-**	0.10	-
*Homo* sp. (60%)	76	0.24	157,680	**-**	**-**	0.19

Two different levels of meat consumption were tested for *Homo* sp. In the first case, meat represents 30% of the energy intake, while in the second case, meat represents up to 60% of energy intake.

### Comparing different scenarios and conditions

**Figure 4:**
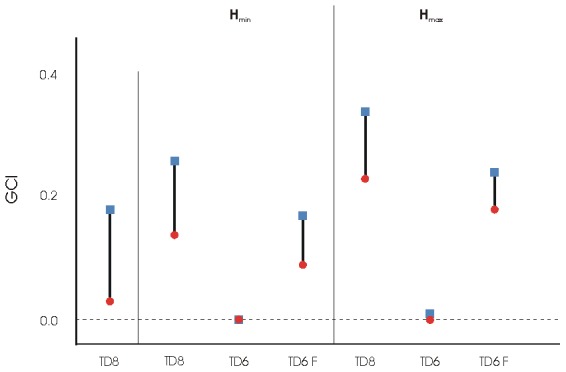
Graphical representation of the global competition index (GCI) for different scenarios. H_min_ denotes the scenarios that include human presence and assume low animal resource requirements for the human population, while H_max_ denotes the scenarios that include human presence and assume high animal resource requirements. TD8: TD8 assemblage; TD6: TD6-2 assemblage. TD6 F: TD6-2 assemblage plus *Homotherium latidens*. GCI takes values from 0 to 1, 0 being minimum competition and 1 being maximum competition. The solid circles represent the condition of maximum total available biomass (TAB-M). The solid squares represent the condition of minimum total available biomass (TAB-m). The black bars represent the range of values between TAB-m and TAB-M.

Two scenarios were considered for TD8: only the species in the TD8 assemblage and the TD8 assemblage plus *Homo* sp. In addition, we considered two other factors: TAB (TAB-m or TAB-M) and *Homo* sp. requirements (H_min_ or H_max_). Thus, we had six different scenarios for TD8 (Table S2 and Figure 4). Comparing the scenarios, we found the worst case to be the hypothetical assemblage of TD8 with *Homo* sp. meeting 60% of its energetic requirements from meat (H_max_) and TAB at a minimum (TAB-m). The scenario with less competition between secondary consumers occurs when TAB is at a maximum (TAB-M). As might be expected, the parameter with the greatest effect is TAB (Table 6 and Figure 4).

**Table 6:** Global competition index (GCI) for TD8 and TD6-2 assemblages and several hypothetical scenarios.

	TAB-m	TAB-M
TD8	0.19	0.04
TD8 H_min_	0.27	0.14
TD6 H_min_	0.00	0.00
TD6 F H_min_	0.17	0.09
TD8 H_max_	0.35	0.23
TD6 H_max_	0.01	0.00
TD6 F H_max_	0.24	0.18

TAB-m: minimum total available biomass; TAB-M: maximum total available biomass; H_min_: meat represents 30% of the energy intake; H_max_: meat represents 60% of the energy intake; F: scenario with a large felid such as *Homotherium latidens*.

### Distribution of resources

The distribution of resources in all scenarios yield viable ecosystems with regard to the minimun viable population density (MVPD) of the species involved, estimated using equations provided by Silva and Downing [108] (Table 6 and Figure 4). Nevertheless, it should be noted that the MVPD corresponds to populations living in extreme conditions and prone to extinction because MVPD is estimated on the basis of populations classified as “endangered”, “vulnerable”, or “close to extinction” by the IUCN [109].

With respect to the energy requirements met, mainly the species that satisfies a smaller percentage of its energy requirement in all cases is *Panthera gombaszoegensis* while *Lynx* sp. is the least affected by the competition for resources (Table S2). Even with the inclusion of *Homo* sp. all species reach viable densities, slightly higher when *Homo* sp. has minor requirements. We used as reference density for *Homo* sp. 0.24 individuals per square kilometre, the highest density observed in recent hunter-gatherers (Table 2). In the worst case, scenario with *Homo* sp. and TAB-m condition, *Panthera gombaszoegensis* met 49% of their requirements with 0.06 individuals per square kilometre; *Canis mosbachensis*, *Ursus* sp. and *Hyaena* sp. met 71% of their requirements with 0.24, 0.03 and 0.09 individuals per square kilometre, respectively; *Crocuta crocuta* met 61% of its requirements with a density of 0.08 individuals per square kilometre; and *Lynx* sp. met 81% of its requirements with a density of 0.38 individuals per square kilometre. *Homo* sp. met 60% of its requirements with a density of 0.15 individuals per square kilometre for TAB-m (see SCI in Table S2).

### Comparison between TD8 and TD6-2

We take the conditions for the human groups inhabiting Atapuerca 900,000 years ago, represented by the TD6-2 assemblage, as a reference against which resource availability and competition between carnivores in TD8 may be compared (Figure 4). TAB varies in TD6-2 from a minimum of 457,693 kcal/km^2^*year to a maximum of 583,638 kcal/km^2^*year (see Table 3 in [57]), in both of which are below the respective values observed for TD8 (496,659 and 643,201 kcal/km^2^*year, respectively). Moreover, in the TD6-2 assemblage, the maximum available resources (TAB-M) are higher than the maximum consumer requirements (TDB-M = 461,028 kcal/km2*year when *Homo antecessor* consumes more meat) and thus, all species are able to fulfil their requirements. Meat resources were high enough at TD6-2 to sustain the expected densities, even in excess (Table S3), except in the case of the most unfavourable scenario with TAB-m and the diet of *Homo antecessor* including a high amount of meat. Conversely, in the case of the TD8 assemblage, this does not occur, and TAB-M (643,201 kcal/km^2^*year) is insufficient to satisfy the secondary consumers' requirements (TBD is equal to 689,279 kcal/km^2^*year). Despite that TAB being lower in TD6-2 than in TD8, TD6-2-TAB meets the carnivores' requirements and TD8-TAB does not. This implies a higher degree of competition between secondary consumers in TD8 than in TD6-2. When the global competition indices are compared (Figure 4), we found that TD8 had a higher GCI than TD6-2, whether *Homo antecessor* is assumed to be highly dependent on animal resources (H_max_) or not (H_min_) for TAB-M condition but not for H_max_ with TAB-m. In addition, we evaluated two hypothetical scenarios: the TD6-2 assemblage with a large felid such as *Homotherium latidens* [60] and TD8 with *Homo* sp. In the hypothetical scenario of the TD6-2 assemblage with *Homotherium latidens* and TAB-m, TD8-GCI is similar to TD6-2-GCI with *Homo antecessor* consuming H_min_. When *Homo antecessor* is considered to be more dependent on meat resources (H_max_), TD6-2-GCI is slightly higher than TD8-GCI. However, if *Homo* sp. is included in the TD8 assemblage, both for H_min_ and H_max_ scenarios, competition increases to a level higher than observed in TD6-2 with *Homotherium latidens* (Figure 4 and Table 6). As it might be expected, the highest competition is observed when *Homo* is included in the TD8 assemblage with high meat consumption requirements (H_max_). Under these conditions, GCI is 0.35 for TAB-m and greater than 0.2 for TAB-M (Figure 4 and Table 6). It is worth noting that the inclusion of *Homo* sp. and *Homotherium latidens* in TD8 and TD6-2 levels softens the differences between both macromammal guilds.

## Discussion

Evidence of human presence in Europe during the period 0.5–0.7 Ma is very scarce. Although several European sites provide abundant evidence of human presence before and after this period, the European continent was apparently depopulated during the 0.5–0.7 Ma interval. The results presented here suggest that during this period, the environment was more hostile to a hominin population than it was previously. The Atapuerca environment was able to sustain the expected population densities of a diverse carnivore guild, including *Homo antecessor*, 0.9 Ma, as shown by the application of the model to the TD6-2 assemblage, even with the inclusion of a large felid not recorded in the fossil assemblage. In contrast, the higher values of the global competition index (GCI) obtained for TD8 in comparison to TD6-2 suggest an environment with a higher intraguild competition for resources at Atapuerca 0.6 Ma. Consequently, survival opportunities for *Homo* at Atapuerca would have been higher in the late Early Pleistocene than in the early Middle Pleistocene. Certainly the value of the population density obtained for *Homo* at TD8 is higher than the minimum viable population size (MVPS) for these species, according to the equation provided by Silva and Downing [108]. Nevertheless, the value of MVPD should be considered an absolute minimum and not an average for a sustainable population. For this reason, we focus our interpretation on the relative values of GCI in concluding that the conditions for a human population were worse at TD8 than at TD6-2 and that the Atapuerca environment was less suitable for a permanent human settlement at 0.6 Ma than at 0.9 Ma. These results support the hypothesis that the human absence from TD8 was related to a more hostile environment during this period at Atapuerca, characterised by a higher competition and less access to resources than in the previous period. These results support the interpretation of Blain et al. [110] that the Atapuerca area was not necessarily always a suitable place for human settlement.

The differences between TD6-2 and TD8 are due to different community structures. Cuenca-Bescós and García [111] and Cuenca-Bescós et al. [112] differentiate several faunal units (FU) in Gran Dolina and separate TD6-2 and TD8 assemblages into two different faunal units FU4 and FU5, respectively. FU4 is characterised by the presence of *Homo antecessor* and the large red-toothed shrew *Dolinasorex glyphodon*, and FU5 is considered the local range zone of *Microtus ratticepoide*s and *Blanus cinereus* [25], [111], [112]. With respect to large primary consumer mammals, it can be observed that the differences between TD6-2 and TD8 are reflected in *Castor fiber* and *Mammuthus* sp. which do not appear in the TD8 assemblage, and *Macaca* sp., *Megaloceros solilhacu*s and *Hippopotamus* sp., which do not appear in TD6-2 assemblage. The rest of the species are equal in both assemblages. As *Castor fiber* and *Mammuthus* sp. are approximately equivalent in terms of biomass to *Macaca* sp. and *Hippopotamus* sp., respectively (because these species belong to the same body mass categories) the main change between both assemblages is the presence of *Megaloceros solilhacus*. This species provides biomass to the categories of 45 to 90 kg, 180 to 360 kg and 360 to 1,000 kg. As shown in Figures 3A and 3B, the patterns of biomass output distribution are different in both assemblages. In the TD8 assemblage, the fourth (180–360 kg) and fifth (360–1,000 kg) categories contain higher proportion of biomass than in TD6-2. TD8 presents a more balanced biomass distribution than TD6-2, with a trapezoidal form rather than a triangular form.

The differences between TD6-2 and TD8 are larger for the secondary consumer group. *Canis mosbachensis*, *Crocuta crocuta*, *Lynx* sp. and *Ursus* sp. (*Ursus dolinensis* to TD6-2 level) are present in both assemblages. The TD8 level marks the last occurrence of *Canis mosbachensis* and *Crocuta crocuta* in the Gran Dolina sequence [112]. The main difference between TD8 and TD6-2 is the absence of *Homo antecessor* and the presence of *Panthera gombaszoegensis* and *Hyaena* sp. in the younger assemblage. *Panthera gombaszoegensis* is recorded at Atapuerca in the lower levels of Gran Dolina in TD3-TD4 and TD5 and also in Sima del Elefante in TE9 and TE12, all of which are older than 0.7 Ma [25]. If *Homo antecessor* were considered an effective hunter, the main difference between the TD6-2 and TD8 palaeoecosystems would be the presence of the scavenger *Hyaena* sp. In addition, it may be assumed that Middle Pleistocene human groups also used scavenging as a feeding strategy and were competitors of *Hyaena* sp., which might also be considered a partial ecological equivalent of *Homo*. In this interpretation, *Homo* would be replaced in the TD8 assemblage by two competitors: *Hyaena* sp. and *Panthera gombaszoegensis*. Alternatively, the absence of *Homo* may be interpreted as a key factor in determining the structure of the TD8 carnivore guild, allowing the presence of certain species that would be competitively displaced if a human population, absent for reasons other than competition, were present in the area. In this alternative interpretation, the absence of human settlement at Atapuerca 0.6 Ma would not be the consequence but the cause of the community structure observed in the TD6-2 assemblage.

Although the results presented here suggest more adverse conditions in TD8 than in TD6-2 other possible explanations for the absence of hominins at TD8 should be considered. The Gran Dolina cave and its sedimentation dynamics constituted a favourable environment for the establishment of carnivore dens and shelters 0.6 Ma [66], but may not have been favourable for human access, unlike TD6-2, because the entrance into the cave was smaller during the deposition of TD8 [66]. The possible effect of an accumulation bias in the TD8 fossil assemblage against certain species should also be considered because the main accumulation agents were hyaenas [66]. Proboscideans were a common faunal element in the Iberian Peninsula during the late Early, Middle and Late Pleistocene [113], but their remains are rarely transported to dens and carnivore shelters, except maybe in the case of *Homotherium* [114], [115]. However, because the secondary consumers present at TD8 were not able to prey on proboscideans, their presence at Atapuerca 0.6 Ma would have had little effect on the intensity of competition and thus would not affect our results. The hypothetical presence of the sabertooth *Homotherium latidens*, a large predator that has been recorded at Gran Dolina in level TD5 (Early Pleistocene) and in level TD10-3 (Middle Pleistocene) [25] would have significantly increased intraguild competition. Given that saberthooths were likely able to kill juvenile, and very old proboscideans [55], [57], if both *Homotherium latidens* and a proboscidean species were present at Atapuerca 0.9 Ma, biomass output would have been increased in the heavier body mass categories, but little of this additional biomass could have been consumed by predators. The presence of a proboscidean would increase resource availability carrion, which would have reduced competition among scavengers. Conversely, competition between predators would be increased by the presence of a top predator like *Homotherium*. Thus, the presence of these species at Atapuerca in the early Middle Pleistocene would change the values of the results presented here, but not their meaning nor our interpretation.

With respect to the debate about the continuity or discontinuity of the peopling of Europe in the early Middle Pleistocene, the results presented here show that a discontinuity in hominin occupation existed at Gran Dolina that coincided with an increase in carnivore competition. According to this interpretation, at approximately 0.6 Ma the TD8 palaeoecosystem was not as favourable to maintaining a hominin settlement as it was at 0.9 Ma, and Atapuerca had no conditions that made it favourable as refuge for human populations, thus representing a “bottleneck” [49] to the human population at the Sierra de Atapuerca. Finlayson et al. [116] propose that *Homo* preferred mosaic landscapes and that homogeneous habitats were ignored by humans throughout the Palaeolithic. Although the Palaeolithic human groups were able to adapt to a wide range of environments [117], they required heterogeneous habitats [116]. If the Atapuerca palaeoecosystem were more homogenous 0.6 than 0.9 Ma, the absence of hominins at TD8 could be explained by their inability to settle homogeneous environments. With respect to vegetation, and according to Rodríguez et al. [25], TD8 presents a high dominance of Mediterranean taxa, while in TD6-2, Mediterranean taxa occur together with cold and dry adapted species, suggesting the existence of a mosaic habitat with areas of steppe vegetation. The preference of *Homo* for heterogeneous environments could indicate its inability to directly compete against specialist species, like open habitat cursorial predators [118], and its need for high environmental diversity to obtain resources using a generalist strategy.

It is likely, environmental conditions for *Homo* were better 0.9 Ma than in later periods, due to higher resource availability and less competition with other carnivores. A faunal turnover occurred in Europe at the beginning of the Middle Pleistocene [4], [54], [58], marked by the appearance of new carnivores and increased herbivore richness [4], [57], that changed the structure of mammalian communities throughout Europe.

This dramatic change in the ecological scenario at the beginning of the Middle Pleistocene might be linked to the appearance in Europe of behavioral and technological improvements and innovations that increased the hunting abilities and survival opportunities of hominins. The use of fire for food processing improved the palatability and edibility of foods and increased energy gain [119]. Interestingly, both technology and hominin populations were completely different before and after the critical interval of the alleged European depopulation. Nevertheless, the subsistence strategies of human groups were successful in both the Early and Middle Pleistocene [120], [121]. Roebroeks [67] suggests a demographic population increase and a human expansion in the Middle Pleistocene on the basis of the technological improvements that occurred during this time.

It is difficult to point towards a unique explanation for the absence of humans from TD8, but what can be said is that survival opportunities for a human population were worse at TD8 than at TD6-2 because intraguild competition was higher. Nevertheless, it is necessary to take into account that the excavation of TD8 affected an area of only 24 m^2^ [122]. It is expected that future excavation of a larger area of this level will provide more faunal remains and other evidence to shed light on the hominin population continuity or discontinuity debate.

The structure of the mammalian palaeocommunity and the intensity of competition in the predatory guild likely constrained human ecodynamics in the Pleistocene. This study provides a new avenue for evaluating the relative suitability of a palaeoenvironment for a human hunter-gatherer population, taking into consideration resource availability and competition intensity. Fossil assemblages with and without evidence of human presence may be compared providing a different way to approach the study of variations in human presence in the Palaeolithic. Although this approach does not provide information about the mechanisms that may have produced changes from one community structure to another, it quantifies changes in trophic relationships using the global competition index (GCI). Investigation of more European sites using a similar approach will help to test whether the apparent discontinuity in the peopling of Europe is related to more adverse environmental conditions or decreased survival opportunities at the beginning of the Middle Pleistocene (0.7−0.5 Ma), as suggested by the Atapuerca record, or whether other mechanisms are implicated.

## Conclusions

The reconstruction of trophic relationships in palaeocommunities and the comparison of the intensities of intraguild competition, using the global competition index (GCI) presented here make it possible to evaluate the relative suitability of past ecosystems for hominins. Increased intraguild competition made conditions at Atapuerca more adverse for secondary consumers at 0.6 than 0.9 Ma. This increased competition is a possible cause of the apparent depopulation of the Atapuerca area in the early Middle Pleistocene. Nevertheless, more sites of this period should be studied with a similar approach to determine whether increased intraguild competition for resources played a role in changes in the distribution of *Homo* in Europe in the 0.5–0.7 Ma interval. Both sites with and without evidence of human presence should be compared to shed light on the fiercely debated question of the continuity *vs.* discontinuity of human occupation in Europe at the beginning of the Middle Pleistocene. This study demonstrates that mathematical modeling is a helpful tool in addressing this topic.

## Supporting Information

Table S1 **Specimen Number.**
(XLSX)

Table S2 **Sustainable densities of the TD8 secondary consumers for six different scenarios (see text) for maximum and minimum total available biomass (TAB).** Estimated density of carnivores (individuals per square kilometre), nutritional requirements (kilocalories per year), total intake (kilocalories per year), unsatisfied requirements (kilocalories per year), sustainable density (individuals per square kilometre), Species Competition Index (SCI). Total intake is defined as the biomass (in kcal) consumed by the species after dividing TAB among the secondary consumers, taking into account the distribution of TAB by body size category (Table 4) and the carnivore preferences (Table 3).
(DOCX)

Table S3 **Sustainable densities of TD6-2 secondary consumers for eight different scenarios (see text) for maximum and minimum total available biomass (TAB).** Estimated density of carnivores (individuals per square kilometre), nutritional requirements (kilocalories per year), total intake (kilocalories per year), unsatisfied requirements (kilocalories per year), sustainable density (individuals per square kilometre), Species Competition Index (SCI), Total intake is defined as the biomass (in kcal) consumed by the species after dividing TAB among the secondary consumers, taking into account the distribution of TAB by body size category (Table 4) and the carnivore preferences (Table 3).
(DOCX)
