# Supplementary material for: Discontinuity of Human Presence at Atapuerca during the Early Middle Pleistocene: A Matter of Ecological Competition?
Source: PLoS One. 2014 Jul 23;9(7):e101938. doi: 10.1371/journal.pone.0101938 (PMC4114206; doi:10.1371/journal.pone.0101938)
Supplement: Table S2 — Sustainable densities of the TD8 secondary consumers for six different scenarios (see text) for maximum and minimum total available biomass (TAB). Estimated density of carnivores (individuals per square kilometre), nutritional requirements (kilocalories per year), total intake (kilocalories per year), unsatisfied requirements (kilocalories per year), sustainable density (individuals per square kilometre), Species Competition Index (SCI). Total intake is defined as the biomass (in kcal) consumed by the species after dividing TAB among the secondary consumers, taking into account the distribution of TAB by body size category (Table 4) and the carnivore preferences (Table 3). (DOCX) [file pone.0101938.s002.docx]

Table S2. Sustainable densities of the TD8 secondary consumers for six different scenarios (see text) for maximum and minimum total available biomass (TAB).

| Scenario | Species | Density (ind/km2) | Requirements (kcal/year) | Total Intake (kcal/year) | Not satisfied requirements (kcal/year) | Sustainable Density (ind/km2) | SCI |
| --- | --- | --- | --- | --- | --- | --- | --- |
| **TD8** |  |  |  |  |  |  |  |
| Minimum TAB | |  |  |  |  |  |  |
|  | *Canis mosbachensis* | 0.42 | 40,062 | 29,300 | 10,762 | 0.30 | 0.27 |
|  | *Crocuta crocuta* | 0.13 | 217,864 | 169,425 | 48,439 | 0.10 | 0.22 |
|  | *Lynx* sp. | 0.47 | 19,825 | 19,573 | 252 | 0.46 | 0.01 |
|  | *Ursus dolinensis* | 0.06 | 23,970 | 17,531 | 6,439 | 0.04 | 0.27 |
|  | *Panthera gombaszoegensis* | 0.11 | 224,627 | 141,669 | 82,957 | 0.07 | 0.37 |
|  | *Hyaena* sp. | 0.17 | 162,932 | 119,162 | 43,770 | 0.12 | 0.27 |
| Maximum TAB | |  |  |  |  |  |  |
|  | *Canis mosbachensis* | 0.42 | 40,062 | 38,553 | 1,510 | 0.39 | 0.04 |
|  | *Crocuta crocuta* | 0.13 | 217,864 | 209,400 | 8,464 | 0.12 | 0.04 |
|  | *Lynx* sp. | 0.47 | 19,825 | 19,724 | 100 | 0.46 | 0.01 |
|  | *Ursus dolinensis* | 0.06 | 23,970 | 23,067 | 903 | 0.05 | 0.04 |
|  | *Panthera gombaszoegensis* | 0.11 | 224,627 | 193,901 | 30,726 | 0.10 | 0.14 |
|  | *Hyaena* sp. | 0.17 | 162,932 | 156,793 | 6,139 | 0.16 | 0.04 |
| **With *Homo* sp.** | |  |  |  |  |  |  |
| Minimum TAB | |  |  |  |  |  |  |
| **High Hunter** | |  |  |  |  |  |  |
|  | *Canis mosbachensis* | 0.42 | 40,062 | 23.652 | 16.410 | 0,25 | 0,41 |
|  | *Crocuta crocuta* | 0.13 | 217,864 | 133.680 | 84.184 | 0.08 | 0.39 |
|  | *Lynx* sp. | 0.47 | 19,825 | 16.018 | 3.806 | 0.38 | 0,19 |
|  | *Ursus dolinensis* | 0.06 | 23,970 | 14.151 | 9.819 | 0.03 | 0,41 |
|  | *Panthera gombaszoegensis* | 0.11 | 224,627 | 114.568 | 110.059 | 0.06 | 0,49 |
|  | *Hyaena* sp. | 0.17 | 162,932 | 96.191 | 66.741 | 0.10 | 0,41 |
|  | *Homo* sp, | 0.24 | 157,680 | 98.399 | 59.281 | 0.15 | 0.38 |
| **Low Hunter** | |  |  |  |  |  |  |
|  | *Canis mosbachensis* | 0.42 | 40,062 | 26.159 | 13.903 | 0.27 | 0.35 |
|  | *Crocuta crocuta* | 0.13 | 217,864 | 150.134 | 67.730 | 0.09 | 0.31 |
|  | *Lynx* sp. | 0.47 | 19,825 | 18.339 | 1.486 | 0.43 | 0.07 |
|  | *Ursus dolinensis* | 0.06 | 23,970 | 15.651 | 8.319 | 0.04 | 0.35 |
|  | *Panthera gombaszoegensis* | 0.11 | 224,627 | 124.796 | 99.830 | 0.06 | 0.44 |
|  | *Hyaena* sp. | 0.17 | 162,932 | 106.388 | 56.545 | 0.11 | 0.35 |
|  | *Homo* sp. | 0.24 | 78,840 | 55.193 | 23.647 | 0.17 | 0.30 |
| Maximum TAB | |  |  |  |  |  |  |
| **High Hunter** | |  |  |  |  |  |  |
|  | *Canis mosbachensis* | 0.42 | 40,062 | 31.784 | 8.278 | 0.33 | 0.21 |
|  | *Crocuta crocuta* | 0.13 | 217,864 | 164.814 | 53.050 | 0.10 | 0.24 |
|  | *Lynx* sp. | 0.47 | 19,825 | 15.070 | 4.755 | 0.36 | 0.24 |
|  | *Ursus dolinensis* | 0.06 | 23,970 | 19.017 | 4.953 | 0.04 | 0.21 |
|  | *Panthera gombaszoegensis* | 0.11 | 224,627 | 161.380 | 63.246 | 0.08 | 0.28 |
|  | *Hyaena* sp. | 0.17 | 162,932 | 129.265 | 33.668 | 0.13 | 0.21 |
|  | *Homo* sp. | 0.24 | 157,680 | 121.872 | 35.808 | 0.19 | 0.23 |
| **Low Hunter** | |  |  |  |  |  |  |
|  | *Canis mosbachensis* | 0.42 | 40,062 | 35.139 | 4.924 | 0.37 | 0.12 |
|  | *Crocuta crocuta* | 0.13 | 217,864 | 181.972 | 35.891 | 0.11 | 0.16 |
|  | *Lynx* sp. | 0.47 | 19,825 | 17.194 | 2.631 | 0.41 | 0.13 |
|  | *Ursus dolinensis* | 0.06 | 23,970 | 21.024 | 2.946 | 0.05 | 0.12 |
|  | *Panthera gombaszoegensis* | 0.11 | 224,627 | 177.767 | 46.860 | 0.09 | 0.20 |
|  | *Hyaena* sp. | 0.17 | 162,932 | 142.908 | 20.025 | 0.15 | 0.12 |
|  | *Homo* sp. | 0.24 | 78,840 | 67.198 | 11.642 | 0.20 | 0.15 |
|  |  |  |  |  |  |  |  |

Estimated density of carnivores (individuals per square kilometre), nutritional requirements (kilocalories per year), total intake (kilocalories per year), unsatisfied requirements (kilocalories per year), sustainable density (individuals per square kilometre), Species Competition Index (SCI). Total intake is defined as the biomass (in kcal) consumed by the species after dividing TAB among the secondary consumers, taking into account the distribution of TAB by body size category (Table 4) and the carnivore preferences (Table 3).
