# Supplementary material for: Discontinuity of Human Presence at Atapuerca during the Early Middle Pleistocene: A Matter of Ecological Competition?
Source: PLoS One. 2014 Jul 23;9(7):e101938. doi: 10.1371/journal.pone.0101938 (PMC4114206; doi:10.1371/journal.pone.0101938)
Supplement: Table S3 — Sustainable densities of TD6-2 secondary consumers for eight different scenarios (see text) for maximum and minimum total available biomass (TAB). Estimated density of carnivores (individuals per square kilometre), nutritional requirements (kilocalories per year), total intake (kilocalories per year), unsatisfied requirements (kilocalories per year), sustainable density (individuals per square kilometre), Species Competition Index (SCI), Total intake is defined as the biomass (in kcal) consumed by the species after dividing TAB among the secondary consumers, taking into account the distribution of TAB by body size category (Table 4) and the carnivore preferences (Table 3). (DOCX) [file pone.0101938.s003.docx]

Table S3. Sustainable densities of TD6-2 secondary consumers for eight different scenarios (see text) for maximum and minimum total available biomass (TAB).

| Scenario | Species | Density (ind/km2) | Requirements (kcal/year) | Total Intake (kcal/year) | Not satisfied requirements (kcal/year) | Sustainable Density (ind/km^2^) | | SCI | |
| --- | --- | --- | --- | --- | --- | --- | --- | --- | --- |
| **TD6-2** |  |  |  |  |  |  | |  | |
| Minimum TAB | |  |  |  |  |  | |  | |
| High Hunter | |  |  |  |  |  | |  | |
|  | *Canis mosbachensis* | 0.34 | 40,808 | 40,276 | 532 | 0.33 | 0.01 | |  |
|  | *Crocuta crocuta* | 0.13 | 217,864 | 216,092 | 1,772 | 0.13 | 0.01 | |  |
|  | *Lynx* sp, | 0.30 | 20,621 | 20,621 | 0 | 0.30 | 0.00 | |  |
|  | *Ursus dolinensis* | 0.05 | 24,054 | 23,740 | 314 | 0.05 | 0.01 | |  |
|  | *Homo antecessor* | 0.24 | 157,680 | 156,072 | 1,608 | 0.24 | 0.01 | |  |
| Low Hunter | |  |  |  |  |  | |  | |
|  | *Canis mosbachensis* | 0.34 | 40,808 | 40,808 | 0 | 0.34 | | 0.00 | |
|  | *Crocuta crocuta* | 0.13 | 217,864 | 217,864 | 0 | 0.13 | | 0.00 | |
|  | *Lynx* sp, | 0.30 | 20,621 | 20,621 | 0 | 0.30 | | 0.00 | |
|  | *Ursus dolinensis* | 0.05 | 24,054 | 24,054 | 0 | 0.05 | | 0.00 | |
|  | *Homo antecessor* | 0.24 | 78,840 | 157,680 | 0 | 0.24 | | 0.00 | |
| Maximum TAB | |  |  |  |  |  | |  | |
| High Hunter | |  |  |  |  |  | |  | |
|  | *Canis mosbachensis* | 0.34 | 40,808 | 40,808 | 0 | 0.34 | | 0.00 | |
|  | *Crocuta crocuta* | 0.13 | 217,864 | 217,864 | 0 | 0.13 | | 0.00 | |
|  | *Lynx* sp, | 0.30 | 20,621 | 20,621 | 0 | 0.30 | | 0.00 | |
|  | *Ursus dolinensis* | 0.05 | 24,054 | 24,054 | 0 | 0.05 | | 0.00 | |
|  | *Homo antecessor* | 0.24 | 157,680 | 157,680 | 0 | 0.24 | | 0.00 | |
| Low Hunter | |  |  |  |  |  | |  | |
|  | *Canis mosbachensis* | 0.34 | 40,808 | 40,808 | 0 | 0.34 | | 0.00 | |
|  | *Crocuta crocuta* | 0.13 | 217,864 | 217,864 | 0 | 0.13 | | 0.00 | |
|  | *Lynx* sp, | 0.30 | 20,621 | 20,621 | 0 | 0.30 | | 0.00 | |
|  | *Ursus dolinensis* | 0.05 | 24,054 | 24,054 | 0 | 0.05 | | 0.00 | |
|  | *Homo antecessor* | 0.24 | 78,840 | 78,840 | 0 | 0.24 | | 0.00 | |
| **TD6 with *Homotherium latidens*** | |  |  |  |  |  | |  | |
| Minimum TAB | |  |  |  |  |  | |  | |
| High Hunter | |  |  |  |  |  | |  | |
|  | *Canis mosbachensis* | 0.34 | 40,808 | 27,155 | 13,654 | 0.23 | | 0.33 | |
|  | *Crocuta crocuta* | 0.13 | 217,864 | 163,664 | 54,200 | 0.10 | | 0.25 | |
|  | *Lynx* sp, | 0.30 | 20,621 | 19,803 | 819 | 0.29 | | 0.04 | |
|  | *Ursus dolinensis* | 0.05 | 24,054 | 16,006 | 8,048 | 0.04 | | 0.33 | |
|  | *Homo antecessor* | 0.24 | 157,680 | 115,297 | 42,383 | 0.18 | | 0.27 | |
|  | *Homotherium latidens* | 0.07 | 234,164 | 115,768 | 118,396 | 0.04 | | 0.51 | |
| Low Hunter | |  |  |  |  |  | |  | |
|  | *Canis mosbachensis* | 0.34 | 40,808 | 34,753 | 6,055 | 0.29 | | 0.15 | |
|  | *Crocuta crocuta* | 0.13 | 217,864 | 197,227 | 20,637 | 0.12 | | 0.09 | |
|  | *Lynx* sp, | 0.30 | 20,621 | 14,882 | 5,739 | 0.22 | | 0.28 | |
|  | *Ursus dolinensis* | 0.05 | 24,054 | 20,485 | 3,569 | 0.05 | | 0.15 | |
|  | *Homo antecessor* | 0.24 | 78,840 | 139,127 | 18,553 | 0.21 | | 0.12 | |
|  | *Homotherium latidens* | 0.07 | 234,164 | 177,164 | 57,000 | 0.05 | | 0.24 | |
| Maximum TAB | |  |  |  |  |  | |  | |
| High Hunter |  |  |  |  |  |  | |  | |
|  | *Canis mosbachensis* | 0.34 | 40,808 | 34,753 | 6,055 | 0.29 | | 0.15 | |
|  | *Crocuta crocuta* | 0.13 | 217,864 | 197,227 | 20,637 | 0.12 | | 0.09 | |
|  | *Lynx* sp, | 0.30 | 20,621 | 14,882 | 5,739 | 0.22 | | 0.28 | |
|  | *Ursus dolinensis* | 0.05 | 24,054 | 20,485 | 3,569 | 0.05 | | 0.15 | |
|  | *Homo antecessor* | 0.24 | 157,680 | 139,127 | 18,553 | 0.21 | | 0.12 | |
|  | *Homotherium latidens* | 0.07 | 234,164 | 177,164 | 57,000 | 0.05 | | 0.24 | |
| Low Hunter |  |  |  |  |  |  | |  | |
|  | *Canis mosbachensis* | 0.34 | 40,808 | 38,143 | 2,666 | 0.32 | | 0.07 | |
|  | *Crocuta crocuta* | 0.13 | 217,864 | 211,793 | 6,071 | 0.13 | | 0.03 | |
|  | *Lynx* sp, | 0.30 | 20,621 | 16,775 | 3,846 | 0.24 | | 0.19 | |
|  | *Ursus dolinensis* | 0.05 | 24,054 | 22,483 | 1,571 | 0.05 | | 0.07 | |
|  | *Homo antecessor* | 0.24 | 78,840 | 75,832 | 3,008 | 0.23 | | 0.04 | |
|  | *Homotherium latidens* | 0.07 | 234,164 | 192,817 | 41,347 | 0.06 | | 0.18 | |

Estimated density of carnivores (individuals per square kilometre), nutritional requirements (kilocalories per year), total intake (kilocalories per year), unsatisfied requirements (kilocalories per year), sustainable density (individuals per square kilometre), Species Competition Index (SCI), Total intake is defined as the biomass (in kcal) consumed by the species after dividing TAB among the secondary consumers, taking into account the distribution of TAB by body size category (Table 4) and the carnivore preferences (Table 3).
